# Quality of Drug Allergy Documentation in a Resource-Limited Paper-Based Hospital in Pakistan: Audit of Concordance and Completeness

**DOI:** 10.3390/healthcare14070957

**Published:** 2026-04-06

**Authors:** Akef Obeidat, Athar Ud Din, Muhammad Amir Khan, Amara Asad Khan, Eshal Atif, Muhammad Atif Mazhar, Muhammad Zain Khan, Sadia Qazi

**Affiliations:** 1 Department of Anatomy, College of Medicine, Alfaisal University, Riyadh 11533, Saudi Arabia; aobeidat@alfaisal.edu; 2 Department of Internal Medicine, MTI Mardan Medical Complex, Mardan 23200, Pakistan; atharuddin11298@gmail.com (A.U.D.); dramirkhan95@gmail.com (M.A.K.); 3 Rashid Hospital, Dubai P.O. Box 7272, United Arab Emirates; asadaliamara@gmail.com; 4 College of Medicine, Alfaisal University, Riyadh 11533, Saudi Arabia; eatif@alfaisal.edu; 5 Department of Pharmacy, Abasyn University, Ring Road (Charsadda Link), Peshawar 25000, Pakistan; khan54zayn@gmail.com

**Keywords:** drug allergy documentation, allergy audit, medication safety, paper-based records, clinical notes, drug Kardex, inter-system concordance, Cohen’s kappa, completeness, patient safety, Pakistan, quality improvement, SQUIRE

## Abstract

**Highlights:**

**What are the main findings?**

- Drug allergy documentation showed very poor concordance between the two paper-based systems (Cohen’s κ = 0.0079).
- Allergy status was documented in the drug Kardex for 94.3% of patients, but in clinical notes for only 25.0%, a 69.3 percentage-point gap; discordant pairs showed that non-documentation was far more likely in clinical notes (discordant-pair OR = 62.00).
- Among patients with a history of allergy, 0% met the five-element completeness standards; entries were typically generic and lacked key details (drug name, reaction type, severity, date, and treatment).

**What are the implications of the main findings?**

- The Kardex-only pattern (70.5%) indicates a specific, actionable failure point in which allergy data exist but are not reliably transferred to clinical notes for prescribing decisions.
- In resource-limited paper-based settings, low-cost measures, such as structured templates, prompts to check the Kardex, and interdisciplinary communication protocols, may help reduce documentation gaps without requiring full electronic systems.

**Abstract:**

**Background/Objectives:** Accurate drug allergy documentation is essential for patient safety; however, documentation quality remains poor worldwide. In resource-limited settings that rely on paper records, allergy information may become fragmented across multiple forms, and evidence on concordance between paper-based documentation systems is limited. This audit assessed concordance between clinical notes and drug Kardex records, and completeness of drug allergy documentation entries, in a manual hospital system. **Methods:** This retrospective clinical audit, reported in accordance with SQUIRE 2.0 guidelines, examined 88 randomly selected patient records from 525 consecutive admissions to a general medicine ward in Pakistan during June–July 2024, retrospectively reviewed in August 2024. The audit assessed allergy status documentation in clinical notes and the drug Kardex, evaluated completeness against five internationally recommended elements (drug name, reaction description, severity, date, and treatment), and measured inter-system concordance using McNemar’s test and Cohen’s kappa. **Results:** Drug allergy status was documented in 25.0% of clinical notes (95% CI: 16.5–35.4%) versus 94.3% of drug Kardex records (95% CI: 87.2–98.1%), representing a 69.3 percentage-point gap (McNemar χ^2^ = 59.06, *p* < 0.001). Inter-system agreement was poor (κ = 0.0079; 95% CI: −0.046 to 0.062), with an overall concordance of 28.4%. Discordant pairs showed that undocumented allergy status was far more likely in clinical notes than in the drug Kardex (OR = 62.00). Kardex-only documentation occurred in 62 of 88 patients (70.5%). Among nine patients with documented allergy history in at least one source, none met the five-element completeness standards (0%; 95% CI: 0.0–33.6%). Recorded entries were generic statements such as “drug allergy” or “allergic to antibiotics” without clinically actionable details. **Conclusions:** Drug allergy documentation showed two major quality failures: poor concordance between parallel paper records and lack of actionable detail in recorded entries. The two systems functioned independently rather than as complementary safety checks, with allergy information often present in the drug Kardex but absent from clinical notes. This Kardex-only failure mode may be a practical target for quality improvement through structured five-element templates, prompts for clinicians to review the drug Kardex, and interdisciplinary allergy-reconciliation workflows. These strategies require prospective evaluation in this setting.

## 1. Introduction

Drug allergy documentation, adverse drug reactions (ADRs), and allergy labeling are related but conceptually distinct. ADR is any harmful unintended response to a medication at a normally used dose. Drug allergy refers specifically to immune-mediated reactions (a subset of ADRs), whether confirmed or suspected through clinical assessment [1]. Allergy documentation is the practice of recording allergy history, reaction details, and associated clinical information in the medical record, independent of whether the allergy has been formally verified. This distinction matters because documentation quality can fail independently of whether the patient’s allergy status is accurately known; a patient may have a well-characterized reaction history that is nonetheless recorded incompletely, generically, or not at all.

Adverse drug reactions affect approximately 8.3% of primary care patients, with nearly one-quarter considered preventable [2]. Among hospitalized older adults, ADRs account for 3.3% to 23.1% of admissions [3]. When allergy documentation is absent, generic, or siloed in a location not accessible during prescribing, clinicians cannot assess contraindication risk, identify cross-reactive agents, or make informed decisions about first-line therapy alternatives [1,4,5]. The consequences include both from unnecessary avoidance of effective drugs, which is a well-documented problem with penicillin-allergy labels [6,7], and re-exposure to causative agents in patients whose histories are simply not visible at the point of care. Accurate, complete, and accessible allergy documentation is therefore, a foundational patient-safety requirement in any prescribing environment.

International guidelines from the World Health Organization, the National Institute for Health and Care Excellence (NICE), and allergy practice groups specify five documentation elements required for safe prescribing: specific drug name, reaction description, severity classification, date or timeframe of occurrence, and treatment received [2,8,9]. Each serves a distinct clinical function: the drug name enables contraindication checking and cross-reactivity assessment; the reaction description distinguishes immune-mediated from intolerance responses; the severity classification informs the urgency of avoidance; the date or timeframe contextualizes whether the reaction occurred under conditions that are still clinically relevant; and the treatment received indicates the seriousness of the event at the time. Without all five elements, a documented allergy entry may be present in the record but clinically uninterpretable. Despite this guidance, audits consistently report low baseline completeness across multiple settings [10,11,12].

Documentation quality varies considerably across healthcare settings. Large-scale studies have shown substantial variability in free-text allergy entries, including multiple spellings for the same allergen, which weakens clinical decision support [10]. High rates of drug allergy alert overrides have been reported, including overrides of severe alerts [13], indicating that documentation problems are not eliminated by digitization alone. In high-income settings, electronic health records (EHRs) with structured fields, prompts, and clinical decision support reduce some documentation gaps [10,14], although inter-source discrepancies persist even within digital systems [14,15]. Quality-improvement interventions combining standard allergy documentation templates, staff education, and workflow redesign have shown meaningful improvements in documentation quality [16,17,18,19]. However, these approaches typically require digital infrastructure and ongoing technical support that are limited in low- and middle-income country contexts.

In many hospitals, including most public facilities in Pakistan, allergy information may become fragmented across multiple paper records, including admission notes, progress notes, consultant entries, and drug Kardex records. These repositories are physically separated and accessed at different times by different clinical teams. From a human factor perspective, this structure creates predictable information transfer failures. The Systems Engineering Initiative for Patient Safety (SEIPS) framework identifies work system design, which includes tool design, task structure, and team communication pathways, as a primary determinant of documentation behavior [20]. When documentation tools are not integrated and no explicit process bridges exist between nursing and physician record streams, allergy data recorded at one point in the workflow may not reach clinicians making prescribing decisions elsewhere. Contributing factors likely include cognitive load during high-volume admissions, interprofessional role perceptions, and hierarchical communication dynamics that shape documentation practices differently across professional groups in low-resource settings [21], as well as the absence of feedback mechanisms that would otherwise signal documentation failures. Whether these parallel paper systems provide true redundancy or function independently is not well understood. The existing evidence on inter-source discrepancies derives largely from electronic systems [14,15], leaving limited evidence on concordance between parallel documentation streams in predominantly paper-based settings.

Paper-based documentation persists across much of Pakistan’s public hospital system because electronic health record adoption faces barriers, including implementation cost, limited digital literacy, unreliable connectivity, and insufficient technical infrastructure [22]. Consequently, clinicians continue to rely on handwritten records as the primary information substrate for prescribing decisions. In Pakistan, poor knowledge of ADR reporting systems and low spontaneous reporting among healthcare professionals have been documented [23], weakening both individual prescribing safety and hospital-level pharmacovigilance capacity. The specific question of which manual documentation source captures allergy information more consistently and whether parallel paper systems transfer information reliably between them has not been quantitatively examined in this context.

We conducted a retrospective clinical audit in the General Medicine ward of the Bacha Khan Medical Complex, Swabi, Pakistan, a tertiary-care facility serving a predominantly rural population using manual paper-based documentation. We assessed 88 randomly selected patient records from 525 consecutive admissions, addressing two prespecified research questions: (1) What is the concordance between clinical notes and drug Kardex records in a manual paper-based documentation system? (2) What proportion of patients with documented allergic reactions meet international completeness standards across all five required documentation elements?

## 2. Methods

### 2.1. Research Design

This retrospective clinical audit (cross-sectional record review) followed the Standards for Quality Improvement Reporting Excellence (SQUIRE) 2.0 guidelines [24,25]. The study was reported in accordance with SQUIRE 2.0; the completed checklist is provided in Supplementary File S1. We used a descriptive cross-sectional design to establish baseline drug allergy documentation quality in a manual paper-based system and provide a foundation for future quality improvement initiatives. The audit was registered with the Quality Improvement Committee of the Bacha Khan Medical Complex (Registration No: BKMC/QI/2024/037) before data collection.

### 2.2. Setting

The audit was conducted in the General Medicine ward of the Bacha Khan Medical Complex in Swabi, Pakistan, a tertiary-care teaching hospital serving the Khyber Pakhtunkhwa province. The facility uses a manual paper-based documentation system in which clinical notes (admission clerking, progress notes, consultant notes, and discharge summaries) and drug Kardex records (medication administration records maintained by nursing staff) serve as the primary repositories of patient information; no electronic health record system was in use during the study period. Ethics approval was obtained in August 2023. The audit retrospectively reviewed records of patients admitted during June–July 2024 (the clinical reference period), capturing routine clinical practice without concurrent interventions. Record extraction was conducted in August 2024, after the clinical reference period had closed. The ward operates with a typical nurse-to-patient ratio of 1:8 and experiences a high patient turnover, with approximately 250 monthly admissions. No prior quality improvement initiatives have focused on allergy documentation at this institution.

### 2.3. Participants

The source population comprised 525 consecutive patients admitted to the General Medicine ward between June and July 2024. The sample size was calculated using standard formulas for single-proportion estimates [26], assuming a 50% prevalence of complete documentation, a 95% confidence level, and a 9.5% margin of error, requiring a minimum of 88 records. Patient records were selected using simple random sampling: a computer-generated random number sequence was applied to all 525 ward admission register medical record numbers, and the first 88 unique numbers drawn constituted the sampling frame. The records were retrieved by the audit team using these numbers without substitution.

The inclusion criteria were as follows: (1) age ≥ 18 years, (2) admission to the general medicine ward during June–July 2024, (3) hospital stay ≥ 24 h, and (4) availability of complete medical records, including both clinical notes sections and drug Kardex. The exclusion criteria were as follows: (1) incomplete records with missing clinical notes or Kardex sections; (2) illegible handwriting preventing reliable extraction; (3) transfers from other wards or hospitals; and (4) discharge against medical advice within 24 h. All 88 randomly selected records met the inclusion criteria. It should be noted that the requirement for complete records in both documentation systems may have excluded the most disorganized or incomplete files, which could represent a best-case scenario and potentially underestimate the true prevalence of documentation failure in the ward population; this is addressed further in the limitations section. The final sample comprised 42 men (47.7%) and 46 women (52.3%).

### 2.4. Instruments

The audit tool was developed in three stages based on international standards from the NICE guideline CG183 [9], AHRQ resources [27], and the WHO Medication Without Harm initiative [8]. In the first stage, a draft item pool was generated from these three source documents, mapping each documentation element to its corresponding international guidance. In the second stage, the draft tool was independently reviewed by two senior physicians and two registered nurses for face validity, clinical feasibility, and clarity of coding instructions; feedback was incorporated before finalization. In the third stage, the tool was pilot-tested on 10 records (excluded from the final analysis) to assess extraction consistency and identify ambiguous items. The finalized tool comprised three sections: (A) patient demographics (age, gender, and admission date), (B) documentation presence (clinical notes vs. drug Kardex records), and (C) documentation quality (five required elements for patients with a known allergy history).

Inter-rater reliability was assessed through independent parallel review of 10 randomly selected records by three auditors, yielding Cohen’s kappa coefficients of 0.85–0.92 (mean κ = 0.89), indicating excellent agreement [28].

### 2.5. Operational Definitions

Operational definitions were established prior to data collection to ensure consistent coding of allergy-related information across paper-based sources. These definitions distinguished clinically actionable allergy status documentation from vague or nonspecific mentions and were used to guide both auditor training and subsequent concordance analyses.

Drug allergy status documented: Any explicit statement regarding allergy status (e.g., “NKDA,” “allergic to penicillin,” “denies drug allergies”). Generic statements such as “drug allergies present,” “allergic,” or “drug allergy” written without specifying the causative agent were systematically coded as allergy status not documented for the purposes of concordance analysis, as they do not constitute actionable clinical information. This coding rule was applied consistently across both clinical notes and drug Kardex records and was specified in the auditor training materials prior to data collection.

Clinical notes: All handwritten entries, including admission clerking, daily progress notes, consultant notes, and discharge summaries. Documentation was considered present if allergy status appeared in any section.

Drug Kardex: Medication administration record maintained by nursing staff (typically at bedside or nursing station), containing prescribed medications and a designated allergy documentation section. The Kardex allergy field was reviewed for any entry, including no known drug allergy (NKDA) statements, and named allergy entries.

### 2.6. Procedures

Data collection occurred between 1–31 August 2024, after the clinical reference period (June–July 2024) had closed. The audit team included one senior medical officer (with 5 years’ experience) and two medical residents, who were independent of the clinical teams providing care during June and July 2024. Before data collection, the auditors completed a standardized two-hour training session covering audit objectives, tool use, extraction methods, and coding rules for ambiguous documentation, including the treatment of allergy statements, as described in Section 2.5, and confidentiality procedures. The auditors independently reviewed three sample records during training to standardize coding interpretation and resolve any disagreements before live data collection began.

Each record was systematically reviewed using a structured tool. Auditors verified inclusion criteria, examined clinical notes from admission to discharge for allergy documentation, and reviewed drug Kardex record documentation. For patients with a documented allergy history, auditors extracted all five required elements. Data were recorded in a standardized spreadsheet with predefined fields, drop-down menus for categorical variables, and validation rules (e.g., binary restriction to yes/no and duplicate ID checks). The file was password-protected and stored on an encrypted institutional server accessible only to the audit team.

For quality control, 10% of the completed records (*n* = 9) were subject to an independent re-audit by a second reviewer blinded to the initial findings. No discrepancies were identified. Data completeness was verified using automated checks for missing values prior to analysis.

### 2.7. Data Analysis

Descriptive statistics (frequencies, percentages) were computed for categorical variables; age was summarized as the mean ± standard deviation. Documentation rates were reported with 95% confidence intervals calculated using the Wilson score interval method [29]. All analyses were performed using IBM SPSS Statistics for Windows, version 29.0 (IBM Corp., Armonk, NY, USA).

For RQ1 (concordance), documentation presence in clinical notes and drug Kardex was compared in a 2 × 2 contingency table. McNemar’s test evaluated paired differences in documentation proportions between the two systems [30]. The discordant-pair odds ratio (OR) was calculated as the ratio of discordant pairs in which clinical notes were undocumented and the Kardex was documented (cell b) to the reverse (cell c), following standard McNemar paired-analysis procedures [30]. Agreement between systems was quantified using Cohen’s kappa (κ), interpreted as: <0.20 poor, 0.21–0.40 fair, 0.41–0.60 moderate, 0.61–0.80 substantial, and 0.81–1.00 almost perfect agreement [28]. Because documentation rates were highly asymmetric between systems (a condition known to suppress kappa independently of true agreement through prevalence bias) [31], overall concordance (the proportion of records with matching documentation status across both systems) was also reported alongside kappa as a supplementary agreement measure.

For RQ2 (completeness), analyses were restricted to records with documented allergy history in at least one source. For each of the five predefined documentation elements (specific drug name, reaction description/type, severity classification, date/timeframe of occurrence, and treatment provided), frequencies and proportions were calculated. Overall completeness was defined as documentation of all five elements. For element-level reporting, the primary denominator was the subset of patients with allergy entries recorded specifically in the drug Kardex, consistent with its role as the primary allergy documentation repository in this setting; the overall completeness rate and sensitivity analysis used all patients with allergy documentation in any source as the denominator. Because the completeness subgroup was small, 95% confidence intervals were calculated using the exact binomial (Clopper–Pearson) method. All tests were two-tailed with significance set at *p* < 0.05.

### 2.8. Ethical Considerations

Ethical clearance was granted by the Office of the Chairman Ethical Review Board, MTI-GKMC/BKMC Swabi, Khyber Pakhtunkhwa, Pakistan (F. No. 2269/Ethical Board/GKMC; dated 16 August 2023). A waiver of informed consent was obtained because the study involved retrospective review of existing records with no patient contact and minimal risk. The linkage key was destroyed after data validation. Data access was restricted to the audit team, all of whom were independent of the clinical teams responsible for patient care during the June–July 2024 reference period. No member of the ward’s treating team participated in data extraction.

## 3. Results

### 3.1. Sample Characteristics and Overall Documentation

We analyzed 88 patient records from consecutive admissions during June–July 2024. The sample included 42 men (47.7%) and 46 women (52.3%). Median length of stay was 4 days (IQR: 3–6; range: 2–14). The most common admission diagnoses were infectious diseases (32.0%, *n* = 28), cardiovascular disorders (23.9%, *n* = 21), and respiratory conditions (18.2%, *n* = 16). Inter-rater reliability was excellent across the three auditors (Cohen’s κ range: 0.85–0.92; mean κ = 0.89), and blinded re-audit of 10% of records revealed no discrepancies.

Drug allergy status was documented more consistently in drug Kardex than in clinical notes. The Kardex contained allergy status for 83 of 88 patients (94.3%; 95% CI: 87.2–98.1); this figure includes both documented allergic reactions and explicitly recorded negative allergy status (NKDA or equivalent notation indicating no known drug allergy). Clinical notes documented status for only 22 patients (25.0%; 95% CI: 16.5–35.4), representing a 69.3 percentage-point gap (McNemar χ^2^ = 59.06, *p* < 0.001). At least one system documented allergy status for 84 patients (95.5%), but only 21 patients (23.9%) had documentation in both systems. Four patients (4.5%) had no allergy documentation in either location. These findings are summarized in Table 1.

### 3.2. Inter-System Concordance

The two documentation systems functioned largely independently rather than as redundant safety checks. Among the 83 patients with Kardex documentation, only 21 (25.3%) also had documentation in clinical notes, while 62 (74.7%) had Kardex-only documentation. Nearly all patients with clinical note documentation (21 of 22, 95.5%) also had Kardex entries; however, the reverse was uncommon.

**Figure 1:**
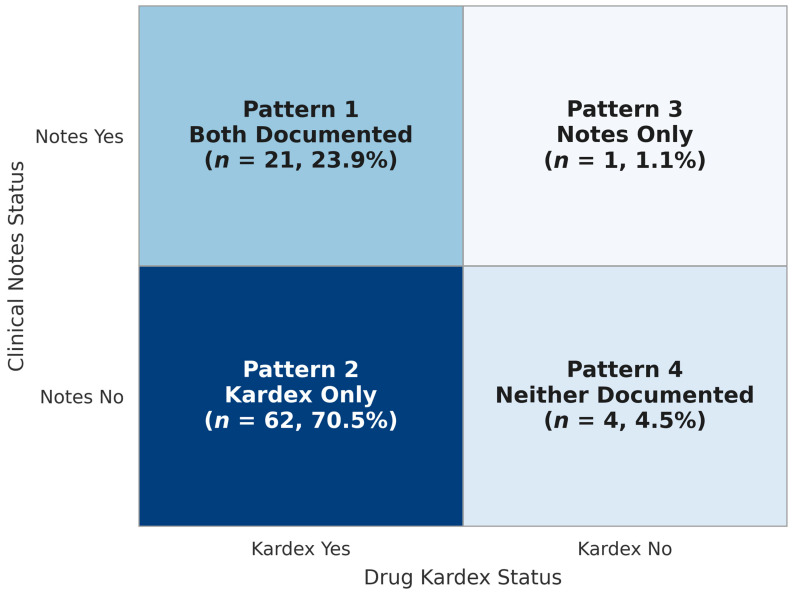
Drug allergy documentation in clinical notes and the drug Kardex (*N*= 88). Each cell shows the number and percentage of patients in that documentation pattern. Kardex-only documentation (*n* = 62, 70.5%) was the dominant pattern.

Statistical agreement between the systems was poor. McNemar’s test confirmed significant asymmetry (χ^2^ = 59.06, *p* < 0.001), and Cohen’s kappa was 0.0079 (95% CI: −0.046 to 0.062), well below the threshold for even fair agreement. The near-zero kappa should be interpreted in the context of highly asymmetric documentation rates (25.0% vs. 94.3%), which can suppress kappa independently of true agreement through prevalence bias [31]; overall concordance (28.4%; 95% CI: 19.4–39.0) is therefore also reported as a supplementary agreement measure. Among discordant-pairs, non-documentation in clinical notes was 62 times more likely than non-documentation in the drug Kardex (OR = 62.00), reflecting the strongly directional nature of the documentation gap: allergy information was systematically absent from clinical notes while present in the Kardex, not randomly missing across both systems. See Table 2 and Figure 1.

### 3.3. Documentation Patterns

We identified four distinct documentation patterns. The dominant pattern was Kardex-only documentation, which occurred in 62 of 88 patients (70.5%; 95% CI: 59.8–79.7). Documentation in both systems occurred in 21 patients (23.9%; 15.6–34.0), while clinical notes-only documentation was rare (1 patient, 1.1%). Four patients (4.5%) had no documentation in either system. Overall, 67 patients (76.1%) showed incomplete redundancy across the two systems. These patterns are summarized in Table 3.

### 3.4. Allergy History and Completeness

Nine patients (10.2%; 95% CI: 4.8–18.6) had a documented allergy history in at least one documentation system. Of these nine patients, six had allergy entries in the Kardex and three had general allergy mentions in clinical notes only (such as “drug allergy”; none of the clinical note entries contained detailed reaction documentation). These nine patients constituted the primary denominator for the completeness analyses (Supplementary Table S1). For concordance analyses, generic non-actionable labels were coded as “status not documented”; for completeness analyses, those same entries were retained as evidence of a recorded allergy history and then assessed for missing detail.

Of the nine patients with any documented allergy history, zero met the five-element completeness standards (0/9; 95% CI: 0.0–33.6%). For element-level reporting, analyses followed the pre-specified primary denominator of six patients with Kardex allergy entries (Section 2.7), consistent with the Kardex’s role as the primary allergy documentation repository in this setting. All entries were generic statements, such as “allergic to antibiotics” or “drug allergy,” without specification of the causative drug, reaction type, severity, date, or treatment received. In a sensitivity analysis restricted to the six patients with Kardex allergy entries specifically, the five-element completeness rate was also 0% (0/6; 95% CI: 0.0–45.9%). No entry contained even a single element beyond a generic label (0/6; 95% CI: 0.0–45.9%), indicating that allergy documentation in this cohort was entirely non-specific across every predefined element. These findings are presented in Table 4. The wider confidence interval for the *n* = 6 subgroup analysis relative to the *n* = 9 analysis reflects the smaller denominator.

### 3.5. Documentation of Negative Allergy Status

Among the 79 patients without a documented allergy history, negative allergy status (NKDA or equivalent) was nearly universal in the Kardex (78/79, 98.7%; 95% CI: 93.1–100.0) but uncommon in clinical notes (19/79, 24.1%; 95% CI: 15.4–34.9). This 74.6 percentage-point difference was statistically significant (McNemar χ^2^ = 56.02, *p* < 0.001), mirroring the pattern observed for positive allergy documentation. The consistency of this pattern across both positive allergy documentation and negative allergy status recording reinforces that the documentation gap is a systemic feature of the clinical note workflow rather than specific to allergy-positive patients. Gender-stratified analyses showed no significant differences in documentation outcomes (Supplementary Table S2). NKDA documentation patterns stratified by gender are presented in Supplementary Table S3.

## 4. Discussion

### 4.1. Principal Findings

This audit of 88 randomly selected patient records from a general medicine ward in Pakistan directly addressed two prespecified research questions. In response to RQ1 (concordance between clinical notes and drug Kardex), inter-system agreement was poor (κ = 0.0079; overall concordance 28.4%), with Kardex-only documentation as the dominant pattern (70.5% of records). Discordant pairs strongly favored Kardex-only documentation (discordant-pair OR = 62.00). In response to RQ2 (five-element completeness among patients with documented allergy history), zero of nine patients with any allergy documentation met international completeness standards (0%; 95% CI: 0.0–33.6%). These findings identify two distinct quality failures, one of documentation visibility and one of documentation content, that operate in parallel and compound each other’s clinical risk.

Documentation visibility refers to whether allergy status is recorded in a location accessible to the prescribing clinician. Documentation completeness refers to whether the recorded entry contains sufficient actionable detail for safe prescribing. In this cohort, both failures were present simultaneously: allergy status was absent from physician-facing clinical notes in 75% of cases (visibility failure), and where allergy entries did exist, none contained a specific drug name, reaction description, severity, date, or treatment received (completeness failure). This distinction is important for intervention design. Visibility failures require workflow and transfer solutions, whereas completeness failures require structured documentation templates and clinician education.

Allergy status was recorded in 25.0% of clinical notes versus 94.3% of drug Kardex records. Kardex-only documentation affected 70.5% of patients, indicating that parallel paper repositories functioned independently rather than as a reliable redundant safety system. Entries were typically generic (e.g., “drug allergy,” “allergic to antibiotics”) without specifying the causative drug, reaction type, severity, timing, or treatment, despite guidance from the World Health Organization, NICE, and AAAAI emphasizing all five elements for safe prescribing [8,9,11].

### 4.2. Comparison with Published Literature

Our findings align with prior audits showing incomplete allergy documentation in diverse settings [32,33]. A 2022 study by Saif et al. in Pakistan similarly found poor concordance between clinical notes and drug Kardex in a cardiology unit, with allergy documentation present in only 34% of case notes compared to 76% in drug charts [34]. Similarly to our study, they identified generic documentation without actionable detail as a major barrier to safe prescribing. The consistency of these findings across different Pakistani hospitals suggests systemic rather than site-specific problems.

The near-total absence of actionable reaction details in our sample mirrors broader international concerns about vague terminology and unstructured allergy entries [32,35]. However, when interpreting comparisons with international literature, it is important to distinguish between studies conducted in electronic systems and those in paper-based environments. Most large-scale concordance studies, including those showing inter-source discrepancies, have been conducted in EHR environments with structured allergy fields, decision-support prompts, and alert systems [10,14,15,36,37]. Our setting had none of these features. The absence of standardized prompts, mandatory fields, and automated feedback mechanisms in paper systems likely contributes to why our κ estimate of 0.0079 was lower than kappa values reported in structured digital settings [37,38], rather than reflecting a qualitatively different problem.

Studies in paper-based or low-resource settings closer to our context, such as the Sudan emergency documentation audit by Mohamed et al. [33] and the contrast hypersensitivity review by Singh et al. [32], have reported similar fragmented documentation patterns, reinforcing that the silo effect identified here is a structural feature of parallel paper workflows rather than an isolated local finding.

The evidence that many penicillin-allergy labels are not confirmed on testing, combined with the broader burden of self-reported antibiotic allergies, reinforces the need for specific and clinically interpretable documentation [6,7].

### 4.3. Why Documentation Fails in Manual Systems

The predominance of Kardex-only documentation suggests information asymmetry: allergy data moves from physician notes to nursing medication records more reliably than in the reverse direction. This pattern is expected in paper workflows in which records are physically separated and accessed at different times by different teams.

The Systems Engineering Initiative for Patient Safety (SEIPS) framework offers a useful theoretical lens for interpreting this pattern [20]. SEIPS identifies work system design encompassing tools, tasks, physical environment, organizational conditions, and team structure as the primary determinants of care process outcomes, including documentation behavior. In the setting described here, clinical notes and drug Kardex are designed as functionally independent tools serving different professional groups, with no structural mechanism requiring information transfer between them. Under SEIPS, the Kardex-only silo is therefore a predictable consequence of work system design rather than individual failure.

Interprofessional dynamics may also contribute. Interprofessional role perceptions and hierarchical communication dynamics in low-resource settings have been shown to create documentation silos, in which nursing and physician teams operate with limited cross-documentation of clinical information [21]. These dynamics may have contributed to the differential documentation patterns observed in this study, when professional groups maintain functionally separate records with no structured reconciliation process. The probability that allergy information documented in one stream will appear in the other is reduced by the structure of the system itself, not necessarily by individual omissions. This mechanism was not directly tested in this study and warrants examination using mixed-methods approaches in future work.

Additional contributing factors include weak ADR reporting culture, limited interdisciplinary communication norms, and the absence of any feedback mechanism that would make documentation failures visible to clinical teams [37,38]. In settings with substantial medication-harm burden and resource constraints, these process weaknesses are amplified [39,40,41].

It is also worth noting that the paper versus digital framing adopted here is a simplification. Many facilities in Pakistan and similar settings operate in hybrid transitional states where some records are electronic and others remain paper-based. Information transfer failures in these environments may be more complex than in purely paper or purely digital systems, as staff navigate multiple parallel documentation tools with unclear ownership boundaries.

### 4.4. Patient Safety Implications

Safety concerns involve both visibility and actionability. When allergy status is absent from physician-facing clinical notes, the prescribing clinician has no signal at the point of decision. When it is present but mentioned as a generic “drug allergy” without a named agent, it is clinically uninterpretable. The clinician cannot determine which drug to avoid, whether cross-reactive agents present a risk, or whether the original reaction was immune-mediated or a less serious intolerance. This creates two distinct downstream harms. The first is re-exposure; a patient with an undocumented or unspecified allergy receives the causative agent, risking anaphylaxis or serious ADR. The second is over-avoidance; a patient labeled “allergic to antibiotics” without further detail is denied potentially optimal first-line agents unnecessarily. Both harms are well-documented in the penicillin allergy literature [6,7] and apply equally to the generic allergy entries identified in this cohort.

Documentation failures in this setting also undermine hospital-level pharmacovigilance capacity. Pharmacovigilance units depend on complete allergy and ADR records to identify drug safety signals, fulfil regulatory reporting obligations to Pakistan’s Drug Regulatory Authority of Pakistan (DRAP), and track institutional ADR burden. When allergy entries consist only of generic labels, they cannot be matched to specific drugs or reaction types, rendering them unusable for surveillance purposes [23]. Improving documentation completeness therefore serves both individual prescribing safety and institutional pharmacovigilance functions.

Given the known burden of adverse drug reactions in the ambulatory and inpatient populations, particularly among older adults [39,40], improving allergy documentation quality directly addresses a prescribing safety risk that is both measurable and modifiable in this setting. Our concordance analysis evaluates the agreement between documentation locations rather than diagnostic accuracy against the true allergy status; the documentation gaps identified represent process failures in recording, not necessarily failures in eliciting allergy history from patients.

### 4.5. Strengths and Limitations

This study followed SQUIRE 2.0 reporting standards [24,25], used random sampling from the ward census, employed pre-specified operational definitions, and achieved excellent inter-rater reliability (mean κ = 0.89) [28]. Appropriate statistical methods were used for paired data and confidence intervals [29,30]. Benchmarking against the WHO, NICE, and AAAAI guidelines supports external interpretability [8,9,11].

The limitations included the single-center scope and moderate sample size, which limited the generalizability. The general medicine ward of a single tertiary facility in Khyber Pakhtunkhwa may not represent documentation practices across Pakistan’s diverse healthcare system, including private hospitals, smaller district facilities, or other clinical departments.

Best-case scenario bias from complete-record inclusion. Inclusion criterion (4) required complete records with both clinical notes and drug Kardex. Records that were incomplete, partially missing, or highly disorganized were excluded. These are precisely the admissions in which documentation failures are most likely to be most severe; therefore, our findings may represent a best-case estimate of the true documentation gap in this setting.

Retrospective abstraction from handwritten records. Handwritten documentation may introduce variability owing to illegibility, inconsistent terminology, and incomplete entries that cannot be fully resolved during the extraction process, representing a source of potential information bias.

Lack of temporal data on documentation timing. The study did not evaluate when allergy information was recorded during the patient care process (at admission, during the ward stay, or at discharge). Timing may influence discrepancies between clinical notes and drug Kardex, and its absence limits mechanistic interpretation.

Mechanism-level explanations unverified. Explanations for the Kardex-only silo, including work system design, interprofessional role perceptions, and cognitive load. These are theoretically grounded but were not tested through staff interviews, direct observation, or process mapping. These remain plausible hypotheses requiring confirmatory mixed methods work.

No linkage to downstream adverse drug events. Documentation patterns were not linked to actual drug re-exposure, preventable ADRs, or other patient outcomes in this cohort. The clinical impact was inferred from the established literature [39,40] rather than directly measured, which is an inherent limitation of the retrospective audit methodology in paper-based systems where outcome data are equally fragmented.

Limited applicability to electronic documentation environments. The findings relate specifically to manual paper-based systems and may not generalize to hospitals that use electronic health records, where structured allergy fields, prompts, and decision support create fundamentally different documentation contexts.

Repeated audit cycles and observation effects. Future audits in this setting should account for the potential Hawthorne effect, whereby awareness of being audited may temporarily improve documentation compliance without producing durable changes [42].

### 4.6. Clinical and Policy Implications

The Kardex-only pattern provides a clear intervention target: improving the transfer of allergy information into physician-facing notes and admission documentation. The following are potential quality improvement strategies that are pending evaluation through prospective intervention studies:

A standardized allergy section in admission templates captures drug name, reaction description, severity, timing, and management received.

Visual reminders at the point of prescribing prompt physicians to consult the drug Kardex before writing medication orders.

A brief structured interdisciplinary handoff checkpoint for allergy reconciliation at the time of admission and shift change

Monthly feedback dashboards generated from brief mini-audits make documentation performance visible to ward teams.

This approach aligns with implementation frameworks that emphasize reach, adoption, and maintenance during scale-up [43]. Local Pakistani evidence demonstrates that low-cost multimodal quality improvement bundles can improve safety-process compliance in resource-limited settings [44]. Audit-and-feedback methods have demonstrated consistent benefits across healthcare contexts [38], and similar structured approaches have succeeded in comparable resource-constrained environments [32,33].

Although staged digitalization remains important in the long term, interim paper-workflow redesign is feasible and should proceed without waiting for full electronic health record deployment [41,44]. It is important to emphasize that these interventions are presented as candidates for structured evaluation and not established solutions. Pre and post-intervention studies with appropriate controls are needed to determine whether documentation improvements achieved through these strategies are durable and translate into measurable reductions in prescribing errors or adverse drug events.

### 4.7. Implications for Research

Future studies should evaluate pre-post intervention effects on Kardex-to-notes transfer and five-element completeness. Time-series audit cycles can test intervention durability while accounting for observation effects [42]. Multi-center studies should examine site-level predictors of better performance, including workflow design, staffing patterns, feedback frequency, and documentation tools. Implementation evaluations should assess reach, adoption, and maintenance using frameworks such as RE-AIM [43].

A critical evidence gap involves linking documentation quality to patient outcomes, such as drug re-exposure, preventable adverse reactions, length of stay, and readmission, in local settings [39,40]. Future work should also examine temporal variations (shift and weekend effects), cross-department reproducibility (medicine versus surgery versus intensive care), and mechanisms through mixed-methods studies with clinician interviews. This would help distinguish knowledge deficits from process-design failures and guide scalable interventions. Future research can also explore structured quality-improvement interventions, such as Lean Six Sigma approaches, which have been associated with reductions in medication errors, improvements in patient safety, and operational efficiency in healthcare settings [45].

Multi-center prospective intervention studies are ultimately needed to determine whether improvements in documentation completeness and inter-system concordance translate into measurable reductions in adverse drug events, prescription errors, and unnecessary drug avoidance in resource-limited settings. Such studies would also establish whether the Kardex-only silo identified here is a consistent feature of manual documentation systems across Pakistan’s public hospital sector or a site-specific finding.

## 5. Conclusions

This audit identified two concurrent documentation failures in manual paper-based hospital systems: a visibility failure, in which allergy status is absent from physician-facing clinical notes in most cases, and a completeness failure, in which allergy entries that did exist lacked an actionable clinical detail across all five required elements.

These findings reflect failures in the recording and transfer of allergy information between parallel documentation streams, not failures in eliciting allergy history from patients or in verifying the true allergy status through clinical assessment. The distinction matters for intervention design: visibility failures require workflow and transfer solutions, whereas completeness failures require structured documentation templates and clinician education.

Meaningful safety improvements may be achievable without full electronic health record deployment. Evidence from Pakistan and similar settings supports the feasibility of low-cost multimodal quality improvement approaches in resource-limited contexts [44], including standardized five-element allergy documentation fields in admission templates, visual reminders prompting physicians to consult the drug Kardex before prescribing, and routine interdisciplinary allergy reconciliation protocols. These strategies require prospective evaluation before their effectiveness in improving documentation completeness and reducing prescribing errors can be established. Multi-center prospective intervention studies are needed to determine whether improvements in documentation quality translate into measurable patient safety gains and whether the patterns identified here are consistent across Pakistan’s broader public hospital sector.

## Figures and Tables

**Table 1:** Drug allergy documentation rates by system (N = 88).

Documentation Site	Documented *n* (%)	Not Documented *n* (%)	95% CI for Documented Rate	Difference from Kardex (Percentage Points, Paired 95% CI)
Clinical notes	22 (25.0)	66 (75.0)	16.5–35.4	−69.3 (−79.5 to −59.2)
Drug Kardex	83 (94.3)	5 (5.7)	87.2–98.1	Reference
Both systems	21 (23.9)	67 (76.1)	15.6–34.0	—
Either system *	84 (95.5)	4 (4.5)	88.8–98.7	—
Neither system	4 (4.5)	84 (95.5)	1.3–11.2	—

Notes: * At least one documentation system (clinical notes and/or drug Kardex) contained the allergy status; —, not applicable.

**Table 2:** Concordance between clinical notes and drug Kardex (*N* = 88).

Clinical Notes Documentation	Kardex Documented *n* (%)	Kardex Not Documented *n* (%)	Row Total *n* (%)	Row Interpretation
Documented	21 (23.9)	1 (1.1)	22 (25.0)	95.5% also in Kardex
Not documented	62 (70.5)	4 (4.5)	66 (75.0)	93.9% Kardex-only
Column total	83 (94.3)	5 (5.7)	88 (100.0)	—
Column interpretation	25.3% also in notes	80.0% both absent	—	—

Notes: Agreement statistics: McNemar χ^2^ = 59.06 (*p* < 0.001); Cohen’s κ = 0.0079 (95% CI: −0.046 to 0.062); discordant-pair OR = 62.00; overall concordance = 25/88 (28.4%; 95% CI: 19.4–39.0); —, not applicable.

**Table 3:** Documentation patterns (N = 88).

Pattern	Description	*n* (%)	95% CI
Pattern 1	Both systems documented	21 (23.9)	15.6–34.0
Pattern 2	Kardex only	62 (70.5)	59.8–79.7
Pattern 3	Clinical notes only	1 (1.1)	0.0–6.2
Pattern 4	Neither system	4 (4.5)	1.3–11.2
Combined 2 + 3 + 4	Incomplete redundancy	67 (76.1)	65.9–84.6

**Table 4:** Five-Element Completeness Among Kardex Allergy Entries (*n* = 6).

Documentation Element	Documented *n* (%)	Not Documented *n* (%)	95% CI for Documented Rate *
Specific drug name	0 (0.0)	6 (100.0)	0.0–45.9
Reaction description/type	0 (0.0)	6 (100.0)	0.0–45.9
Severity classification	0 (0.0)	6 (100.0)	0.0–45.9
Date/timeframe of occurrence	0 (0.0)	6 (100.0)	0.0–45.9
Treatment provided	0 (0.0)	6 (100.0)	0.0–45.9
Complete five-element documentation	0 (0.0)	6 (100.0)	0.0–45.9
Partial documentation (≥1 element beyond generic label)	0 (0.0)	6 (100.0)	0.0–45.9

* Exact binomial (Clopper–Pearson) CI.

## Data Availability

Full raw patient records are not publicly available because they contain potentially identifiable clinical information and are subject to institutional and ethical restrictions. Summary supporting data are provided in the Supplementary Material. A de-identified minimal dataset sufficient to reproduce the main analyses is available from the corresponding author upon reasonable request.

## Supplementary Materials

The following supporting information can be downloaded at: https://www.mdpi.com/article/10.3390/healthcare14070957/s1, Supplementary File S1, Completed SQUIRE 2.0 Checklist; Supplementary Table S1, Allergy-history subgroup characteristics; Supplementary Table S2, documentation outcomes stratified by gender; Supplementary Table S3, NKDA documentation patterns stratified by gender.

## Abbreviations

AAAAI, American Academy of Allergy, Asthma and Immunology; ADR, adverse drug reaction; BKMC, Bacha Khan Medical Complex; CI, confidence interval; EHR, electronic health record; IRB, Institutional Review Board; IQR, interquartile range; MTI, Medical Teaching Institution; NICE, National Institute for Health and Care Excellence; NKDA, no known drug allergy; OR, odds ratio; QI, quality improvement; SEIPS, Systems Engineering Initiative for Patient Safety; SPSS, Statistical Package for the Social Sciences; SQUIRE, Standards for Quality Improvement Reporting Excellence; WHO, World Health Organization; κ, Cohen’s kappa.
