# Supplementary material for: Quality of Drug Allergy Documentation in a Resource-Limited Paper-Based Hospital in Pakistan: Audit of Concordance and Completeness
Source: Healthcare (Basel). 2026 Apr 6;14(7):957. doi: 10.3390/healthcare14070957 (PMC13072779; doi:10.3390/healthcare14070957)
Supplement: Supplementary file 1 [file healthcare-14-00957-s001.zip › healthcare-4181719-supplementary.pdf]

Quality of Drug-Allergy Documentation in a Resource-Limited Paper-Based Hospital in Pakistan: Audit of Concordance and Completeness

Supplementary Material

Supplementary File S1.SQUIRE 2.0 Checklist

Item No.	Section/Item	SQUIRE 2.0 Requirement	Page No.	Manuscript Location
1	Title	Indicate that the manuscript concerns an initiative to improve healthcare	1	Title revised to specify documentation quality, paper-based setting, Pakistan, and audit focus.
2a	Abstract: Search/Index	Provide adequate information to aid in searching and indexing	1	Keywords include drug-allergy documentation, paper-based records, patient safety, Pakistan, quality improvement, and SQUIRE.
2b	Abstract: Structured Summary	Background, local problem, methods, interventions, results, conclusions	1	Structured abstract with Background/Objectives, Methods, Results, and Conclusions.
3	Problem Description	Nature and significance of the local problem	2–3	Introduction outlines patient-safety importance of drug-allergy documentation and risks in paper-based systems.
4	Available Knowledge	Summary of what is currently known, including relevant previous studies	2–4	Introduction reviews WHO/NICE guidance, prior concordance/completeness audits, EHR versus paper documentation, and Pakistan ADR context.
5	Rationale	Frameworks/theories explaining the problem and why intervention(s) expected to work	4	Introduction and Discussion use SEIPS and parallel-documentation workflow concepts; five-element completeness framework specified.
6	Specific Aims	Purpose of the project and of this report	4	Final Introduction paragraph states two prespecified research questions on concordance and completeness.
7	Context	Contextual elements at outset of introducing the intervention	4–5	Setting describes tertiary teaching hospital, paper-based system, ward workload, and absence of prior allergy-documentation QI.
8a	Intervention(s): Description	Description in sufficient detail for reproduction	4	Retrospective baseline audit, registered with the QI committee before data collection.
8b	Intervention(s): Team	Specifics of team involved	6	Procedures describe one senior medical officer and two residents, independent of clinical teams, with standardized training.
9a	Study of Intervention: Approach	Approach for assessing impact	4–7	Retrospective cross-sectional audit; SQUIRE 2.0 compliant; random sampling; structured extraction tool.
9b	Study of Intervention: Causality	Approach to establish outcomes due to intervention	4	Baseline audit design only; causality reserved for future intervention cycles.
10a	Measures: Choice and Rationale	Measures chosen, rationale, operational definitions, validity, reliability	5–7	Five-element completeness framework; operational definitions; pilot-tested tool; inter-rater κ=0.85–0.92.
10b	Measures: Contextual Elements	Ongoing assessment of contextual factors	5, 12–14	Contextual factors discussed in setting and interpreted in Discussion (workload, workflow fragmentation, hybrid systems).
10c	Measures: Data Completeness	Methods for assessing data completeness and accuracy	6–7	10% blinded re-audit; automated completeness checks; spreadsheet validation rules.
11a	Analysis: Methods	Qualitative and quantitative methods for inferences	7	Descriptive statistics; exact/Wilson confidence intervals as reported; McNemar test; Cohen's kappa; discordant-pair OR; contingency tables.
11b	Analysis: Variation	Methods for understanding variation including time effects	7, 11	Gender-stratified supplementary analyses (Supplementary Tables S1 and S3); temporal variation proposed for future studies.
12	Ethical Considerations	Ethical aspects and how addressed, including formal review	7	IRB approval, waiver of consent, de-identification, and restricted data access.
13a	Results: Evolution	Initial steps and evolution over time	8	Two-month audit period (June–July 2024) providing a baseline snapshot.
13b	Results: Details	Process measures and outcomes	8–11	Sections 3.1–3.5: rates (Table 1), concordance (Table 2 and Figure 1),

Item No.	Section/Item	SQUIRE 2.0 Requirement	Page No.	Manuscript Location
				patterns (Table 3), completeness (Table 4), and NKDA documentation (Section 3.5; Supplementary Tables S1–S3).
13c	Results: Context	Contextual elements interacting with intervention	8	Ward characteristics, demographics, admission diagnoses, and length of stay reported.
13d	Results: Associations	Associations between outcomes, intervention, and context	9–11	McNemar asymmetry; discordant-pair OR=62.00; κ=0.0079; Kardex-only pattern 70.5%; 0% five-element completeness.
13e	Results: Unintended Consequences	Unexpected benefits, problems, or costs	N/A	None observed in this baseline audit.
13f	Results: Missing Data	Details about missing data	8	All 88 included records met inclusion criteria; no missing data after quality control.
14a	Summary: Key Findings	Key findings including relevance to aims	12	0% five-element completeness; 69.3-point gap; κ=0.0079; Kardex-only pattern 70.5%; visibility and completeness failures distinguished.
14b	Summary: Strengths	Particular strengths of the project	14	SQUIRE alignment, random sampling, prespecified definitions, high inter-rater reliability, and transparent statistical analysis.
15a	Interpretation: Nature of Association	Nature of association between intervention and outcomes	12–13	Parallel systems functioned independently, producing asymmetric documentation visibility across systems.
15b	Interpretation: Comparison	Comparison with other publications	12–13	Discussion compares findings with Pakistani and international audits and distinguishes paper from structured digital settings.
15c	Interpretation: Impact	Impact on people and systems	13–14	Patient-safety implications include both unsafe re-exposure and unnecessary drug avoidance; pharmacovigilance relevance discussed.
15d	Interpretation: Reasons for Differences	Reasons for observed vs anticipated outcomes	13–14	SEIPS, fragmented workflow, interprofessional role boundaries, hybrid systems, and record separation discussed as plausible but untested mechanisms.
15e	Interpretation: Costs and Trade-offs	Strategic trade-offs including opportunity costs	14–15	Low-cost templates/reminders and reconciliation workflows discussed as interim strategies pending wider digitalization.
16a	Limitations: Generalizability	Limits to generalizability	14	Single-center scope and limited applicability to other wards and fully electronic environments acknowledged.
16b	Limitations: Internal Validity	Confounding, bias, imprecision	14	Best-case selection bias, handwritten-record limitations, and absence of outcome linkage acknowledged.
16c	Limitations: Efforts to Minimize	Efforts to minimize and adjust for limitations	14	Blinded re-audit, inter-rater reliability checks, and prespecified definitions used to reduce bias.
17a	Conclusions: Usefulness	Usefulness of the work	15–16	Clear targets for quality improvement identified in a resource-limited paper-based context.
17b	Conclusions: Sustainability	Sustainability considerations	15	Recurring audit-feedback cycles and scalable low-cost workflow measures discussed.
17c	Conclusions: Spread	Potential for spread to other contexts	15	Applicability to similar low-resource settings and need for multicenter evaluation stated.
17d	Conclusions: Implications	Implications for practice and further study	15–16	Potential QI actions and future research agenda outlined (pre-post, time-series, outcome linkage, mechanism testing).
17e	Conclusions: Next Steps	Suggested next steps	15–16	Prospective intervention studies, mixed-methods work, and multicenter expansion proposed.
18	Funding	Funding sources and role	16	No external funding; APC funded by Alfaisal University.

Supplementary Tables

Supplementary Table S1. Allergy-History Subgroup Characteristics (n=9)

Parameter	n (%)	95% CI
Patients with documented allergy history (overall N=88)	9 (10.2)	4.8–18.6
Male	3 (33.3)	7.5–70.1
Female	6 (66.7)	29.9–92.5
Allergy entries in the Kardex	6 (66.7)	29.9–92.5
General allergy mentions in clinical notes	3 (33.3)	7.5–70.1
Detailed reaction documentation in clinical notes	0 (0.0)	0.0–33.6
Complete five-element documentation (any source)	0 (0.0)	0.0–33.6

Rows use n=9 as the denominator unless otherwise specified. These figures match Section 3.4 of the main manuscript.

Supplementary Table S2. Documentation Outcomes Stratified by Gender

Outcome	Total n (%)	Males (n=42) n (%)	Females (n=46) n (%)	Test	p-value
Clinical notes documented	22 (25.0)	8 (19.0)	14 (30.4)	χ^2	0.218
Drug Kardex documented	83 (94.3)	41 (97.6)	42 (91.3)	Fisher exact	0.363
Both systems documented	21 (23.9)	8 (19.0)	13 (28.3)	χ^2	0.311
Allergy history present	9 (10.2)	3 (7.1)	6 (13.0)	Fisher exact	0.489

No statistically significant sex differences were observed in any documentation outcome.

Supplementary Table S3. NKDA Documentation Patterns Stratified by Gender (n=79 patients without documented allergy history)

Documentation site	Overall documented n (%)	Males (n=39) n (%)	Females (n=40) n (%)	Test	p-value
Drug Kardex	78 (98.7)	38/39 (97.4)	40/40 (100.0)	Fisher exact	0.494
Clinical notes	19 (24.1)	7/39 (17.9)	12/40 (30.0)	χ^2	0.210

Across all 79 patients without documented allergy history, NKDA documentation remained much more frequent in the drug Kardex than in clinical notes (74.6 percentage-point difference; McNemar χ^2=56.02, $p<0.001$).
